# Supplementary material for: A GeV-TeV particle component and the barrier of cosmic-ray sea in the Central Molecular Zone
Source: Nat Commun. 2021 Nov 9;12:6169. doi: 10.1038/s41467-021-26436-z (PMC8578646; doi:10.1038/s41467-021-26436-z)
Supplement: Supplementary file 1 — Supplementary Information [file 41467_2021_26436_MOESM1_ESM.pdf]

A GeV-TeV particle component and the barrier of cosmic-ray sea in the Central Molecular Zone

September 27, 2021

<table>
<tr><th></th><th>Point Sources</th><th>Sagittarius A*</th><th>GCE</th><th>isotropic diffuse background</th><th>ICS</th><th colspan="2">π^0</th></tr>
<tr><td rowspan="2">Fig. 1</td><td rowspan="2">4FGL + 3FHL J1747.2-2822+ 3FHL J1748.6-2816</td><td rowspan="2">point source + power-law spectrum</td><td rowspan="2">Generalized NFW profile + power-law spectrum</td><td rowspan="2">Isotropic profile + power-law spectrum</td><td rowspan="2">GALPROP model A ICS + power-law spectrum</td><td colspan="2">GALPROP model A π^0 + power-law spectrum</td></tr>
<tr><td>CMZ region</td><td>Off-CMZ region</td></tr>
<tr><td rowspan="2">Fig. 1</td><td rowspan="2">4FGL + 3FHL J1747.2-2822+ 3FHL J1748.6-2816</td><td rowspan="2">point source + power-law spectrum</td><td rowspan="2">Generalized NFW profile + power-law spectrum</td><td rowspan="2">isotropic profile + power-law spectrum</td><td rowspan="2">GALPROP model A ICS + power-law spectrum</td><td colspan="2">Planck dust opacity map + power-law spectrum</td></tr>
<tr><td>CMZ region</td><td>Off-CMZ region</td></tr>
<tr><td rowspan="2">Fig. 1</td><td rowspan="2">4FGL + 3FHL J1747.2-2822+ 3FHL J1748.6-2816</td><td rowspan="2">point source + power-law spectrum</td><td rowspan="2"></td><td rowspan="2">isotropic profile + power-law spectrum</td><td rowspan="2">GALPROP model A ICS + power-law spectrum</td><td colspan="2">Planck dust opacity map + power-law spectrum</td></tr>
<tr><td>CMZ region</td><td>Off-CMZ region</td></tr>
<tr><td rowspan="2">Fig. 2</td><td rowspan="2">4FGL(fixed) + 3FHL J1747.2-2822+ 3FHL J1748.6-2816</td><td rowspan="2">point source + power-law spectrum</td><td rowspan="2">Generalized NFW profile + power-law spectrum</td><td rowspan="2">isotropic profile + power-law spectrum</td><td rowspan="2">GALPROP model A ICS + power-law spectrum</td><td colspan="2">Planck dust opacity map + power-law spectrum</td></tr>
<tr><td>CMZ region (index fixed) 3 segments</td><td>Off-CMZ region (index fixed) 8 segments</td></tr>
<tr><td rowspan="2">Fig. 2</td><td rowspan="2">4FGL(fixed) + 3FHL J1747.2-2822+ 3FHL J1748.6-2816</td><td rowspan="2">point source + power-law spectrum</td><td rowspan="2"></td><td rowspan="2">isotropic profile + power-law spectrum</td><td rowspan="2">GALPROP model A ICS + power-law spectrum</td><td colspan="2">Planck dust opacity map + power-law spectrum</td></tr>
<tr><td>CMZ region (index fixed) 3 segments</td><td>Off-CMZ region (index fixed) 8 segments</td></tr>
<tr><td rowspan="2">Fig. 2</td><td rowspan="2">4FGL (fixed) + 3FHL J1747.2-2822 + 3FHL J1748.6-2816</td><td rowspan="2">point source + power-law spectrum</td><td rowspan="2">Generalized NFW profile + power-law spectrum</td><td rowspan="2">isotropic profile + power-law spectrum</td><td rowspan="2">GALPROP model A ICS + power-law spectrum</td><td>CS map + power-law spectrum</td><td>GALPROP model A (total π^0 -H^2 π^0 in the CMZ region) + power-law spectrum</td></tr>
<tr><td>CMZ region (index fixed) 3 segments</td><td>ROI region</td></tr>
<tr><td rowspan="2">Fig. 3 and Fig. 5</td><td rowspan="2">4FGL + 3FHL J1747.2822+ 3FHL J1748.6-2816</td><td rowspan="2">point source + power-law spectrum</td><td rowspan="2">Generalized NFW profile + power-law spectrum</td><td rowspan="2">isotropic profile + power-law spectrum</td><td rowspan="2">GALPROP model A ICS + power-law spectrum</td><td>CS map × $r^{-\alpha}$ and CS map × $r^{-1.35}$ + power-law spectrum</td><td>GALPROP model A (total π^0 -H^2 π^0 in the CMZ region) + power-law spectrum</td></tr>
<tr><td>CMZ region</td><td>ROI region</td></tr>
<tr><td rowspan="2">Fig. 3 and Fig. 5</td><td rowspan="2">4FGL + 3FHL J1747.2-2822+ 3FHL J1748.6-2816</td><td rowspan="2">point source + power-law spectrum</td><td rowspan="2">Generalized NFW profile + power-law spectrum</td><td rowspan="2">isotropic profile + power-law spectrum</td><td rowspan="2">GALPROP model B ICS + power-law spectrum</td><td>CS map × $r^{-\alpha}$ and CS map × $r^{-1.35}$ + power-law spectrum</td><td>GALPROP model B (total π^0 -H^2 π^0 in the CMZ region) + power-law spectrum</td></tr>
<tr><td>CMZ region</td><td>ROI region</td></tr>
<tr><td rowspan="2">Fig. 3 and Fig. 5</td><td rowspan="2">4FGL + 3FHL J1747.2-2822+ 3FHL J1748.6-2816</td><td rowspan="2">point source + power-law spectrum</td><td rowspan="2">Generalized NFW profile + power-law spectrum</td><td rowspan="2">isotropic profile + power-law spectrum</td><td rowspan="2">GALPROP model C ICS + power-law spectrum</td><td>CS map × $r^{-\alpha}$ and CS map × $r^{-1.35}$ + power-law spectrum</td><td>GALPROP model C (total π^0 -H^2 π^0 in the CMZ region) +power-law spectrum</td></tr>
<tr><td>CMZ region</td><td>ROI region</td></tr>
<tr><td rowspan="2">Fig. 3 and Fig. 5</td><td rowspan="2">4FGL + 3FHL J1747.2-2822+ 3FHL J1748.6-2816</td><td rowspan="2">point source + power-law spectrum</td><td rowspan="2"></td><td rowspan="2">isotropic profile + power-law spectrum</td><td rowspan="2">GALPROP model A ICS + power-law spectrum</td><td>CS map × $r^{-\alpha}$ and CS map × $r^{-1.35}$+ power-law spectrum</td><td>GALPROP model A (total π^0 -H^2 π^0 in the CMZ region) + power-law spectrum</td></tr>
<tr><td>CMZ region</td><td>ROI region</td></tr>
<tr><td rowspan="2">Fig. 4</td><td rowspan="2">4FGL + 3FHL J1747.2-2822+ 3FHL J1748.6-2816</td><td rowspan="2">point source + power-law spectrum</td><td rowspan="2">Generalized NFW profile + power-law spectrum</td><td rowspan="2">isotropic profile + power-law spectrum</td><td rowspan="2">GALPROP model A ICS + power-law spectrum</td><td>CS map × $r^{-1.35}$+ power-law spectrum</td><td>GALPROP model A (total π^0 -H^2 π^0 in the CMZ region) + power-law spectrum</td></tr>
<tr><td>CMZ region</td><td>ROI region</td></tr>
<tr><td rowspan="2">Fig. 4</td><td rowspan="2">4FGL + 3FHL J1747.2-2822+ 3FHL J1748.6-2816</td><td rowspan="2">point source + power-law spectrum</td><td rowspan="2">Generalized NFW profile + power-law spectrum</td><td rowspan="2">isotropic profile + power-law spectrum</td><td rowspan="2">GALPROP model A ICS + power-law spectrum</td><td>CS map × r^0 + power-law spectrum</td><td>GALPROP model A (total π^0 -H^2 π^0 in the CMZ region) + power-law spectrum</td></tr>
<tr><td>CMZ region</td><td>ROI region</td></tr>
<tr><td rowspan="2">Fig. 4</td><td rowspan="2">4FGL + 3FHL J1747.2-2822+ 3FHL J1748.6-2816</td><td rowspan="2">point source + power-law spectrum</td><td rowspan="2">Generalized NFW profile + power-law spectrum</td><td rowspan="2">isotropic profile + power-law spectrum</td><td rowspan="2">GALPROP model A ICS + power-law spectrum</td><td>GALPROP model A H^2 π^0 in the CMZ region + power-law spectrum</td><td>GALPROP model A (total π^0 -H^2 π^0 in the CMZ region) + power-law spectrum</td></tr>
<tr><td>CMZ region</td><td>ROI region</td></tr>
<tr><td rowspan="3">Methods: foreground /background effect</td><td rowspan="3">4FGL + 3FHL J1747.2-2822 + 3FHL J1748.6-2816</td><td rowspan="3">point source + power-law spectrum</td><td rowspan="3">Generalized NFW profile + power-law spectrum</td><td rowspan="3">isotropic profile + power-law spectrum</td><td rowspan="3">GALPROP model A ICS + power-law spectrum</td><td colspan="2">GALPROP model A π^0 annulus 2, 3, 4, 5 and 6 + free normalizations</td></tr>
<tr><td colspan="2">GALPROP model A π^0 annulus 1 +power-law spectrum</td></tr>
<tr><td>CMZ region</td><td>Off-CMZ region</td></tr>
<tr><td rowspan="3">Methods: foreground /background effect</td><td rowspan="3">4FGL (fixed) + 3FHL J1747.2-2822 + 3FHL J1748.6-2816</td><td rowspan="3">point source + power-law spectrum</td><td rowspan="3">Generalized NFW profile + power-law spectrum</td><td rowspan="3">isotropic profile + power-law spectrum</td><td rowspan="3">GALPROP model A ICS + power-law spectrum</td><td colspan="2">GALPROP model A π^0 annulus 2, 3, 4, 5 and 6 + free normalizations</td></tr>
<tr><td colspan="2">GALPROP model A π^0 annulus 1 + power-law spectrum</td></tr>
<tr><td>CMZ region (index fixed) 3 segments</td><td>Off-CMZ region (index fixed)</td></tr>
</table>

Supplementary Table 1: Model components used in the likelihood fitting. We fit the data in the

region of interest (ROI), a $10^\circ \times 10^\circ$ square centered on the galactic center, with different components, including point sources from 4FGL catalog [1, 2] and two point sources from 3FHL catalog [3], point source Sagittarius A*, the Galactic Center GeV excess (GCE) modelled by generalized Navarro–Frenk–White (NFW) profile [4], the isotropic diffuse background, the inverse Compton scattering (ICS) component of the Galactic diffuse emission predicted by GALPROP model A , B and C [5] and also the neutral pion decay component (π^0). To investigate the cosmic-ray property in the central molecular zone (CMZ), we single out the CMZ region with different tracers, such as π^0 predicted by GALPROP, Planck dust opacity map [6] and CS map [7], to model the gamma-ray emission from π^0.

References

1 Abdollahi, S. *et al.* Fermi Large Area Telescope Fourth Source Catalog. *Astrophys. J. Supp.* **247**, 33 (2020).

2 Ballet, J., Burnett, T. H., Digel, S. W. & Lott, B. Fermi Large Area Telescope Fourth Source Catalog Data Release 2. *arXiv e-prints* arXiv:2005.11208 (2020).

3 Ajello, M. *et al.* 3FHL: The Third Catalog of Hard Fermi-LAT Sources. *Astrophys. J. Supp*. **232**, 18 (2017).

4 Calore, F., Cholis, I. & Weniger, C. Background model systematics for the Fermi GeV excess. *J. Cosmol. Astropart. Phys.* **2015**, 038 (2015).

5 Ackermann, M. *et al.* The Spectrum of Isotropic Diffuse Gamma-Ray Emission between 100 MeV and 820 GeV. *Astrophys. J.* **799**, 86 (2015).

6 Planck Collaboration *et al.* Planck intermediate results. XLVIII. Disentangling Galactic dust emission and cosmic infrared background anisotropies. *Astron. Astrophys.* **596**, A109 (2016).

7 Tsuboi, M., Handa, T. & Ukita, N. Dense Molecular Clouds in the Galactic Center Region. I. Observations and Data. *Astrophys. J. Supp.* **120**, 1–39 (1999).
